# Replacement with sex steroids in hypopituitary men and women: implications for gender differences in morbidities and mortality

**DOI:** 10.1007/s11154-024-09897-7

**Published:** 2024-10-07

**Authors:** Darran Mc Donald, Tara McDonnell, Michael W. O’Reilly, Mark Sherlock

**Affiliations:** 1 Department of Endocrinology, Beaumont Hospital, Royal College of Surgeons of Ireland, Dublin 9, Dublin, Ireland; 2 Department of Medicine, Royal College of Surgeons in Ireland, Dublin, Ireland

**Keywords:** Hypopituitarism, Gonadotropin deficiency, Hypogonadotropic hypogonadism, Mortality, Sex hormone, Testosterone, Oestrogen

## Abstract

Hypopituitarism is a heterogenous disorder characterised by a deficiency in one or more anterior pituitary hormones. There are marked sex disparities in the morbidity and mortality experienced by patients with hypopituitarism. In women with hypopituitarism, the prevalence of many cardiovascular risk factors, myocardial infarction, stroke and mortality are significantly elevated compared to the general population, however in men, they approach that of the general population. The hypothalamic-pituitary-gonadal axis (HPG) is the most sexually dimorphic pituitary hormone axis. Gonadotropin deficiency is caused by a deficiency of either hypothalamic gonadotropin-releasing hormone (GnRH) or pituitary gonadotropins, namely follicle-stimulating hormone (FSH) and luteinising hormone (LH). HPG axis dysfunction results in oestrogen and testosterone deficiency in women and men, respectively. Replacement of deficient sex hormones is the mainstay of treatment in individuals not seeking fertility. Oestrogen and testosterone replacement in women and men, respectively, have numerous beneficial health impacts. These benefits include improved body composition, enhanced insulin sensitivity, improved atherogenic lipid profiles and increased bone mineral density. Oestrogen replacement in women also reduces the risk of developing type 2 diabetes mellitus. When women and men are considered together, untreated gonadotropin deficiency is independently associated with an increased mortality risk. However, treatment with sex hormone replacement reduces the mortality risk comparable to those with an intact gonadal axis. The reasons for the sex disparities in mortality remain poorly understood. Potential explanations include the reversal of women’s natural survival advantage over men, premature loss of oestrogen’s cardioprotective effect, less aggressive cardiovascular risk factor modification and inadequate oestrogen replacement in women with gonadotropin deficiency. Regrettably, historical inertia and unfounded concerns about the safety of oestrogen replacement in women of reproductive age have impeded the treatment of gonadotropin deficiency.

## Introduction

Hypopituitarism is a heterogeneous disorder characterised by a deficiency in one or more anterior pituitary hormones [1]. It is a rare condition with a prevalence of 375–455 cases per million and an incidence of 20.7–41.1 per million [2, 3]. It is over 30 years since Rosen and Bengtsson published their seminal paper which first demonstrated increased mortality in patients with hypopituitarism [4], this and subsequent studies have confirmed a greater mortality risk in women with hypopituitarism than men [5]. Gonadotropin deficiency (also known as hypogonadotropic hypogonadism) refers to sex hormone deficiency arising from either inadequate secretion or action of hypothalamic gonadotropin-releasing hormone (GnRH) or the pituitary gonadotropins follicle-stimulating hormone (FSH) and luteinising hormone (LH). Gonadotropin deficiency is treated with sex hormone replacement, namely oestrogen (with or without progesterone) in women and testosterone in men. Women with hypopituitarism have a higher burden of comorbidities and mortality risk compared to their male counterparts [5, 6]. This narrative review will outline the differences in comorbidities (cardiovascular risk factors, bone health, hormone-dependent cancers) and mortality risk between sexes. It will then examine the impact of oestrogen (with or without progesterone) and testosterone replacement on these parameters. Finally, we will explore potential explanations for the higher mortality rate experienced by women with hypopituitarism. Given the paucity of evidence on the impact of sex hormone replacement in individuals with gonadotropin deficiency we will also refer to literature on other causes of hypogonadism. Although such evidence cannot be directly extrapolated to pituitary cohorts, it can provide important insights which may help support clinical decision making.

## Aetiology of gonadotropin deficiency

Gonadotropin deficiency is caused by a wide spectrum of disorders as outlined in Table 1. Pituitary adenomas are the most common cause of hypopituitarism, accounting for over half of cases [7]. Adenomas cause gonadotropin deficiency via three distinct mechanisms: mass effect, hormone hypersecretion and treatment of the adenoma [8]. The hypothalamic-pituitary-gonadal axis is particularly susceptible to the suppressive effects of hyperprolactinaemia arising from a prolactinoma or stalk effect. Similarly, hypercortisolism in Cushing’s Disease is frequently associated with hypogonadotropic hypogonadism. Treatment of pituitary adenomas with transsphenoidal surgery and radiotherapy can also result in hypopituitarism [9]. The hierarchy of susceptibility to hormone deficiencies is classically described as growth hormone (GH) followed by gonadotropin, adrenocorticotrophic hormone (ACTH) and finally thyroid-stimulating hormone (TSH) [10]. Other causes of gonadotropin deficiency include craniopharyngiomas [11], hypophysitis [12], traumatic brain injuries [13] and vascular complications such as pituitary apoplexy and subarachnoid haemorrhage [14, 15]. Medications are another important cause of hypopituitarism, the gonadotroph axis is particularly sensitive to the suppressive effects of opiates, anabolic steroids and glucocorticoids [16, 17]. GnRH analogues, used in the treatment of advanced prostate cancer, also induce profound gonadotropin deficiency. Immunotherapy-related hypophysis is emerging as an important cause of hypopituitarism in cancer patients treated with cytotoxic T-lymphocyte associated protein 4 (CTLA-4) or programmed cell death protein 1 (PD-1) inhibitors. However, the gonadotropin axis is less prone to deficiency than the ACTH and TSH axes in these cases [18]. Finally, congenital hypogonadotropic hypogonadism (CHH), is a heterogenous, polygenic disorder characterised by a congenital deficiency of GnRH with otherwise normal pituitary function [19]. This disorder is commonly referred to as Kallman’s syndrome when accompanied by anosmia.

**Table 1:** Aetiology of gonadotropin deficiency

**Acquired Causes**	
**Neoplastic** • Pituitary Adenoma • Craniopharyngioma • Meningioma • Germinoma • Rathke’s Cleft cyst	**Vascular disorders** • Pituitary apoplexy • Subarachnoid Haemorrhage • Sheehan’s Syndrome
**Iatrogenic** • Transsphenoidal surgery • Radiotherapy– brain tumour, pituitary tumour, head/ neck cancer	**Hypophysis** • Lymphocytic • Granulomatous: Sarcoidosis, tuberculosis, ANCA vasculitis • IgG4 • Xanthomatous
**Medications** • Opiates • Glucocorticoids • Immunotherapy: PD-1 & CTLA-4 inhibitors • Gonadotropin hormone releasing hormone (GnRH) analogues • Anabolic Steroids	**Infiltrative** • Langherans Cell Histiocytosis • Haemochromatosis • Sarcoidosis
**Other**	
• Hyperprolactinaemia	• Empty Sella
• Traumatic Brain Injury	
**Congenital Causes**	
Kallmann Syndrome • ANOS-1, FGF-8, CDH7, SOX1, KISSR	
Idiopathic congenital hypogonadotropic hypogonadism	

## Clinical features and diagnosis of gonadotropin deficiency

Gonadotropin deficiency can present with menstrual irregularities (oligomenorrhoea/ amenorrhoea), reduced libido, infertility and dyspareunia in women of reproductive age. Climacteric symptoms including hot flushes, sweats and heat intolerance are reported less frequently in women with gonadotropin deficiency compared to those with primary hypogonadism [20]. Diagnosis relies on low serum oestrogen concentrations, combined with either low or inappropriately normal gonadotropins, after excluding other causes of menstrual dysfunction. The presence of gonadotropin levels not elevated in the postmenopausal range is sufficient to make the diagnosis in post-menopausal women, not on hormone replacement therapy (HRT) [21].

Specific symptoms of gonadotropin deficiency in men include reduced libido, decreased spontaneous erections, diminution of secondary sexual characteristics, gynaecomastia and infertility due to impaired spermatogenesis. Fatigue, low mood, reduced muscle mass and increased adiposity are additional features which cannot be solely ascribed to gonadotropin deficiency. Diagnosis requires two separate early morning, fasting samples, demonstrating low testosterone levels with either low or inappropriately normal gonadotrophins [22]. Additionally, a paired sex hormone-binding globulin is required to accurately interpret testosterone levels as most commercially available testosterone assays measure total testosterone concentrations. CHH, which is 3–5 times more common in men, presents with delayed puberty in adolescence or early adulthood and may be accompanied by anosmia and developmental anomalies such as cleft lip, dental agenesis, ear anomalies, congenital hearing impairment, renal agenesis and bimanual synkinesis [23, 24]. These findings in a patient with confirmed isolated gonadotropin deficiency should prompt genetic screening for CHH.

## Treatment of gonadotropin deficiency

### Treatment of gonadotropin deficiency in women

Sex hormone replacement is the mainstay of treatment in individuals with gonadotropin deficiency not seeking fertility. Notably, the duration of sex hormone replacement differs by gender. Women with gonadotropin deficiency typically receive HRT until the age of natural menopause (and increasingly beyond) whereas men receive testosterone replacement throughout their life [21]. Women with an intact uterus require combined oestrogen and progesterone replacement to prevent endometrial hyperplasia from unopposed oestrogenic stimulation. Progesterone can be administered on a sequential or continuous basis. In sequential regimens, daily oestrogen is combined with progesterone for 12–14 days per month (typically on days 14–28) resulting in a menstrual bleed upon withdrawal, whereas, in continuous regimens, oestrogen and progesterone is administered throughout the month and does not produce bleeding. Continuous regimens are not recommended for women who have had menstrual bleeding in the last 12 months. Women with a prior hysterectomy or progesterone-secreting intra-uterine system can safely use ‘oestrogen-only’ regimens [25]. Amongst available oestrogen formulations, 17β-estradiol (E2) is more physiological than either ethinylestradiol (EE) or conjugated equine oestrogen (CEE). The adequacy of oestrogen replacement should be assessed by evaluating symptoms of oestrogen deficiency and monitoring for side effects. Unlike testosterone replacement in men, there is no biochemical parameter to monitor [21]. Oestrogen can be administered in oral or transdermal (topical gels or patches) preparations, although the latter has several advantages. Firstly, the transdermal route mimics the natural release of oestrogen, avoiding first-past metabolism. Unlike oral oestrogen, it does not induce the synthesis of hepatic proteins including angiotensinogen and coagulation factors, which are key components in the renin-angiotensin-aldosterone system and coagulation cascade, respectively. As a result, transdermal oestrogen is not associated with an increased risk of hypertension, venous thromboembolism and stroke [26–29]. Secondly, oral (but not transdermal) oestrogen antagonises the action of GH at the level of the liver. This antagonism necessitates higher GH replacement doses in women receiving oral oestrogen compared to those on transdermal preparations [30]. Finally, although the combined oral contractive pill (OCP) is the most common form of HRT in women with hypopituitarism [31], it offers unphysiological replacement. It provides pharmacological doses of oestrogen for three weeks followed by one week of oestrogen deficiency when the pill is omitted. Furthermore, the absence of a reliable biomarker to guide oestrogen replacement therapy poses a significant clinical challenge, potentially leading to over-replacement in some women and under-replacement in others. While current guidelines do not offer specific recommendations on HRT preparations in women with hypopituitarism, they should be counselled on the relative risks and benefits of various preparations, enabling them to make informed decisions. Practical guidance for managing gonadotropin deficiency in women is summarised in Table 2.

**Table 2:** Practical guidance for treating gonadotropin deficiency in women

1. Women should receive oestrogen replacement with or without progesterone (depending on the need for endometrial protection) until at least the age of natural menopause.
2. Discuss how the risks and benefits of HRT differ between postmenopausal women and those with gonadotropin deficiency, particularly if women are reluctant to commence hormone replacement.
3. Highlight the beneficial effects of transdermal oestrogen relative to oral, particularly in those co-prescribed GH replacement.
4. Assess the adequacy of oestrogen replacement periodically by monitoring for symptoms of oestrogen deficiency and evidence of over replacement.
5. Undertake periodic assessments of cardiovascular risk factors. Clinicians should be cognisant of the harmful effects of treatment inertia and manage risk factors appropriately, taking into account women with hypopituitarism’s elevated risk of cardiovascular disease.

### Androgen replacement in women with hypopituitarism

Combined gonadotropin and ACTH deficiency results in severe androgen deficiency as production from both the ovaries (testosterone) and adrenal glands (DHEA) is compromised. It has been suggested that androgen deficiency contributes to persistent poor libido, sexual dysfunction and impaired quality of life in women with hypopituitarism despite appropriate hormone replacement. Studies exploring the impact of DHEA replacement on these parameters have yielded conflicting results to date [32]. A meta-analysis reported DHEA led to a modest improvement in quality of life but not sexual functioning in women with primary adrenal insufficiency [33]. Testosterone replacement improved bone mineral density, body composition and sexual functioning in a small study of women with hypopituitarism [34]. However, one-third of the participants in this study also developed features of androgen excess [34]. Guidelines recommend against the routine use of androgen replacement in women with hypopituitarism given the limited efficacy and safety data [35]. In carefully selected women who have persistently impaired quality of life and sexual dysfunction despite optimisation of their pituitary hormone deficits, a trial of androgen replacement may be considered under close supervision. There are currently no systemic androgen preparations licenced for use in women. However, a DHEA pessary was approved by regulatory authorities in the United States and Europe for managing symptoms of vaginal atrophy.

### Treatment of gonadotropin deficiency in men

Gonadotropin deficiency in men not seeking fertility is treated with testosterone replacement [22]. In contrast to women, the adequacy of testosterone replacement is assessed by measuring testosterone concentrations, aiming for levels in the mid-normal range. Various preparations are currently available including intramuscular injections, transdermal gels and oral tablets. Testosterone undecanoate is a long-acting intramuscular depot that is administered every 3 months. It has largely replaced shorter-acting formulations such as testosterone ester, enantate or cypionate. These preparations were administered every 2–4 weeks and subject to peak and trough levels which resulted in fluctuating symptoms. Daily testosterone gels are another option which provides more physiological replacement and ease of dose adjustment [36]. The use of oral testosterone replacement has historically been limited by concerns over hepatotoxicity. However, a new preparation of testosterone undecanoate combined with a novel drug delivery system (Jatenzo^®^) was recently approved. This preparation effectively treats hypogonadism and is not associated with hepatotoxicity as it is absorbed via the lymphatic system [37, 38]. The limited available evidence suggests there is no difference in body composition, cardiovascular parameters and quality of life between the various testosterone formulations [39]. Although intramuscular testosterone injections are associated with greater increases in haematocrit than patch preparations the clinical significance of this finding remains uncertain [40]. Therefore, the choice of testosterone preparation should be based on patient preference, preparation-specific side effects and medication cost and availability.

## Pituitary hormone interactions

Sex hormone replacement can influence the diagnosis and treatment of other pituitary hormone deficiencies. The most clinically relevant interaction relates to the impact of oral oestrogen on the hypothalamic-pituitary-adrenal (HPA) axis. Oral oestrogen increases total cortisol concentrations by up to 67% through stimulating the synthesis of corticosteroid-binding globulin (CBG) [41]. This can mask underlying ACTH deficiency as cortisol assays measure total rather than free (biologically active) cortisol. Oral oestrogen should be withheld for six weeks before assessing the HPA axis to prevent false negative results [42]. Empirical hydrocortisone can be prescribed in the intervening period in those with a moderate to high clinical suspicion of ACTH deficiency. While measurement of free cortisol can circumvent the issue of oestrogen-induced CBG stimulation in women taking the OCP, access to these assays are limited and definitive cut-offs for diagnosing ACTH deficiency have not been established. Early morning salivary cortisone is emerging as a useful tool for evaluating adrenal insufficiency in this setting. The test has a diagnostic accuracy similar to that of the ACTH stimulation test and is unaffected by oral oestrogen as it measures free, rather than total cortisone [43]. Oral oestrogen has a limited impact on the treatment of ACTH deficiency. Increased doses of hydrocortisone are rarely required after initiation of sex hormone replacement. In contrast, higher doses of thyroxine are commonly required as a consequence of starting oral oestrogen [21, 44]. Thyroid function tests should be monitored six to twelve weeks after commencing oral oestrogen to assess the adequacy of thyroxine replacement. Oral but not transdermal oestrogen has a significant impact on the treatment of GH deficiency as it antagonises the action of GH at the level of the liver leading to reduced generation of IGF-1 [45]. This antagonism necessitates higher doses of GH to achieve target insulin-like growth factor 1 (IGF-1) levels. A study by Birzniece et al., estimated GH doses were 50–70% higher in women on oral oestrogen relative to those on transdermal [30]. For these reasons, transdermal oestrogen is preferable to oral in women receiving GH replacement. Testosterone and oral oestrogen replacement have dimorphic effects on GH action. Testosterone replacement in men increases GH secretion, augments GH action, increases IGF-1 production and amplifies the peripheral effects of GH [46]. As a consequence, men are more sensitive to the effects of GH replacement and require lower doses than women [47].

## Comorbidities: cardiovascular risk factors

### Cardiovascular risk factors in hypopituitarism

**Fig. 1:**
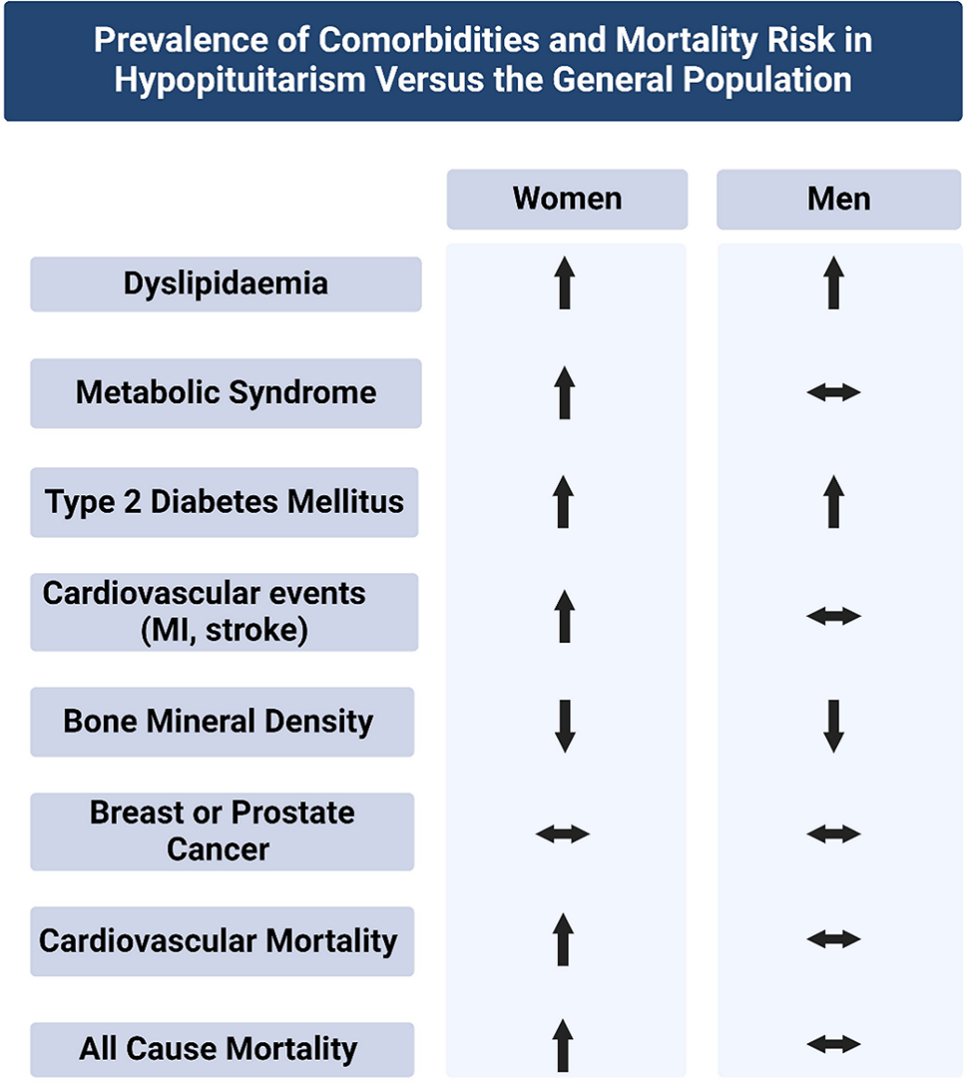
Prevalence of comorbidities in hypopituitarism versus the general population by sex

The prevalence of cardiovascular risk factors including dyslipidaemia, diabetes and metabolic syndrome differs markedly by sex (see Fig. 1). Hypopituitarism is associated with atherogenic lipid profiles, characterised by elevated total cholesterol, low-density lipoproteins (LDL) and triglycerides (TG) and reduced high-density lipoproteins (HDL). In the KIMS database (a GH treatment registry), total cholesterol was elevated in 66% of men and 75% of women, while HDL was below target in 46% of men, 49% of pre-menopausal women and 57% of post-menopausal women before initiating GH replacement [48]. Hypopituitarism is also associated with reduced insulin sensitivity and an increased prevalence of type 2 diabetes mellitus, particularly in women [6, 49]. Similarly, metabolic syndrome (defined by three or more risk factors including obesity, hypertension, fasting hyperglycaemia, hypertriglyceridaemia and reduced HDL) is significantly more common in hypopituitary women, whereas its prevalence in men approaches that of the general population [50]. While findings on the prevalence of hypertension are inconsistent, most studies suggest hypertension is not a significant contributor to cardiovascular disease in patients with hypopituitarism [51]. The higher prevalence of cardiovascular risk factors in women with hypopituitarism relative to men may predispose them to adverse cardiovascular outcomes.

### Cardiovascular risk factors and HRT in women

**Fig. 2:**
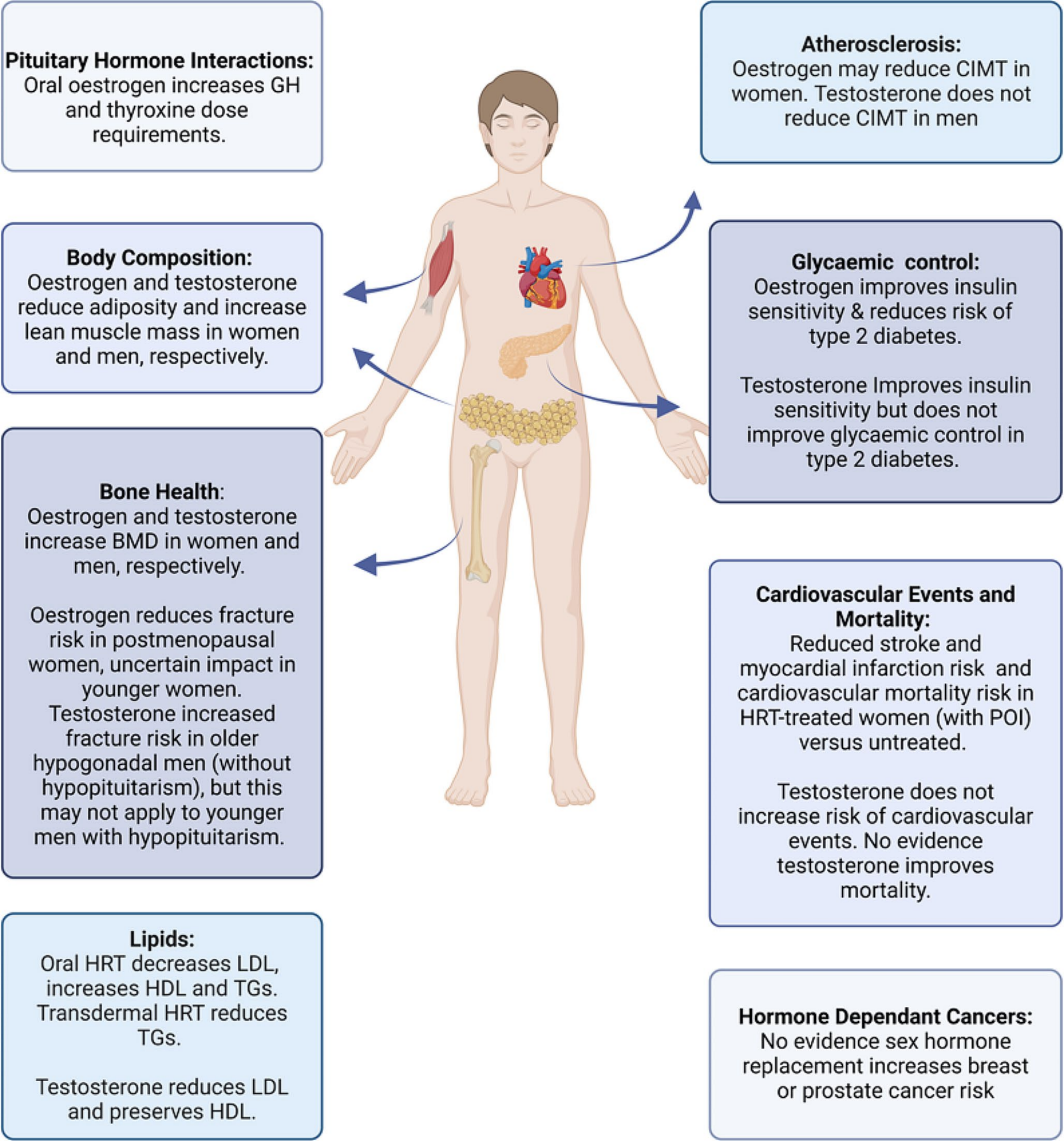
Impact of sex hormone replacement in women and men

Combined and oestrogen-only HRT improves lipid profiles, enhances insulin sensitivity and may even reverse early atherosclerosis (see Fig. 2). Evidence of these beneficial effects is based on studies of women with premature ovarian insufficiency (POI - defined as the development of primary hypogonadism in women under 40 years) and menopause, rather than pituitary cohorts. Oral HRT (combined and oestrogen-only) reduces LDL and raises HDL, although it also increases TGs [52]. Transdermal HRT has the additional benefit of reducing TGs [53]. Studies using hyperinsulinaemic euglycemic clamps have demonstrated combined oral HRT improves insulin sensitivity in post-menopausal women [54]. Trials including the Women’s Health Initiative (WHI) have also reported oral HRT (combined and oestrogen-only) reduces the risk of developing type 2 diabetes [55]. Women with POI experience accelerated atherosclerosis, evident with increased carotid intima-media thickness (CIMT) and subclinical coronary artery disease compared to age-matched, oestrogen-replete controls [56, 57]. The development of atherosclerosis in these women occurs independently of traditional cardiovascular risk factors, highlighting the central role played by premature oestrogen deficiency [58]. Ostberg et al., demonstrated increasing doses of oral oestrogen progressively reduced CIMT, suggesting HRT may reverse early atherosclerosis [59]. While HRT has numerous beneficial metabolic effects in hypogonadal women, this evidence must be extrapolated with caution to those with gonadotropin deficiency given the complex interactions between multiple pituitary hormone deficiencies and their treatments.

### Cardiovascular risk factors and testosterone replacement in men

Testosterone replacement in hypogonadal men has beneficial metabolic effects, however, its impact on the cardiovascular system is complex (see Fig. 2). One study demonstrated body composition, lipid profile, HbA1c and glycaemic control were similar between eugonadal men and those with treated gonadotropin deficiency, suggesting testosterone effectively treats the metabolic abnormalities associated with hypogonadism [39]. It is important to note the complex bidirectional relationship that exists between hypogonadism and visceral adiposity. Hypogonadal individuals have a propensity to develop visceral adiposity and conversely, obesity can lead to hypogonadism. Several mechanisms including suppression of GnRH release, reduced sex hormone-binding globulin and aromatisation of testosterone to oestradiol in men all contribute to obesity-induced hypogonadism. Testosterone has beneficial effects on body composition, increasing muscle mass and strength, while reducing adipose tissue [36, 59]. Testosterone improves lipid profiles by lowering total and LDL cholesterol, however, it can also reduce HDL levels [60]. Testosterone enhances insulin sensitivity although this does not translate into a reduced risk of developing diabetes or improved glycaemic control in men with diabetes [61]. It is important to note that unlike oestrogen replacement in women, testosterone does not improve atherosclerosis (either CIMT or coronary artery plaque) [62, 63]. There was conflicting evidence on the cardiovascular safety of testosterone, prompting the FDA to issue a safety warning in 2014. Concern arose that testosterone’s pro-thrombotic effect may increase the risk of MI following atherosclerotic plaque rupture. The recent TRAVERSE trial reported no increased risk of adverse cardiovascular outcomes in testosterone-treated middle-aged and elderly hypogonadal men [64]. However, there was an increased incidence of pulmonary embolism in the testosterone treated group [64]. It is important to again emphasise these findings may not be generalisable to younger men with hypopituitarism. While testosterone has beneficial metabolic benefits in hypogonadal men, further investigation is required to understand its cardiovascular effects in men with hypopituitarism.

### Cardiovascular risk factor management

Screening for cardiovascular risk factors should be undertaken regularly in patients with hypopituitarism. Despite optimisation of pituitary hormone replacement regimens and lifestyle modification, pharmacological treatment of hyperlipidaemia, hypertension and type 2 diabetes mellitus is frequently required. For example, individuals with hypopituitarism would often require a statin to reach the Endocrine Society guideline LDL target of 1.8mmol/L [65]. Statins are effective at treating dyslipidaemia in patients with hypopituitarism [66]. However, a 2007 sub-group analysis of the KIMS database reported only 5% of patients were treated a statin despite most having elevated LDL levels [67]. Although more recent data on statin prescribing is lacking, our own observations suggest they remain underutilised in patients with hypopituitarism. It is essential patients receive the standard of care for each cardiovascular risk factor while considering their increased risk of cardiovascular disease.

## Bone health

### Bone health in hypopituitarism

Fractures cause significant morbidity, leading to reduced mobility, declines in functional capacity and in the case of hip fractures a substantial mortality risk. Low bone mineral density (BMD) is a measure of bone health which correlates with the risk of fragility fractures. Patients with hypopituitarism have reduced BMD and a 2.7-fold increased risk of fractures relative to the general population [68]. Although the cause of reduced BMD in individuals with hypopituitarism is multifactorial, including GH deficiency and supraphysiological glucocorticoid replacement in ACTH deficient patients, sex hormone deficiency and its treatment play a central role in bone health.

### Bone health and HRT in women

Direct evidence on the impact of oestrogen replacement on bone health in women with gonadotropin deficiency is scarce. One study suggested transdermal combined HRT was more effective at increasing BMD than the OCP in women with functional hypothalamic amenorrhoea [69]. Oral oestrogen reduces IGF-1 levels in individuals receiving GH replacement. Given the trophic effects of IGF-1 on bone [70], this may compromise increases in BMD relative to transdermal HRT. The 7-day break with the OCP also results in periods of oestrogen deficiency, whereas transdermal HRT provides a continuous release of oestrogen. Both combined and oestrogen-only HRT have proven anti-fracture efficacy in postmenopausal women. HRT reduced the risk of osteoporotic fractures by 24% and hip fractures by 33% when compared with placebo in the WHI [71]. However, the anti-fracture efficacy of oestrogen in younger women has not been assessed to date.

### Bone health and testosterone replacement in men

Testosterone replacement enhances several measures of bone quality including increased BMD and improved trabecular bone architecture in both pituitary and non-pituitary, hypogonadal men [72–74]. It was previously postulated testosterone replacement may reduce fracture risk in this cohort. However, recently published data from the Traverse trial unexpectedly reported an increased risk of clinical fractures in hypogonadal men receiving testosterone replacement [75]. It’s likely this finding is attributable to behavioural factors rather than a direct effect of testosterone on bone. As participants in the trial were older and most had age-related or functional hypogonadism, these findings cannot be immediately extrapolated to hypopituitary patients with testosterone deficiency.

## Hormone dependent cancers

### Hormone-dependent cancers in hypopituitarism

Breast and prostate cancer are the most common invasive cancers in women and men, respectively [76, 77]. There is no evidence that patients with hypopituitarism have an increased risk of either cancer. A long-term follow-up study of over 15,000 patients reported standardised incidence ratios of 0.56 (95% CI 0.47–0.77) and 1.31 (95% CI 0.97–1.50) for breast and prostate cancer, respectively [78]. It is important patients with hypopituitarism are counselled and reassured about the impact of sex hormone replacement and cancer risk which we will now discuss.

### HRT and breast cancer

When considering the risk of breast cancer associated with HRT it is essential to differentiate postmenopausal women from those with POI and gonadotropin deficiency. Treatment of the latter represents ‘true’ replacement of premature oestrogen loss whereas it is a pharmacological treatment for climacteric symptoms in postmenopausal women. HRT undoubtedly increases the risk of breast cancer in postmenopausal women [55, 79]. Taking HRT for five years from age 50 increases the risk of breast cancer by approximately 1 in every 50 users of continuous combined HRT; 1 in every 70 users of sequential combined HRT and 1 in every 200 users of oestrogen-only HRT [80]. In contrast, there is no evidence to suggest HRT increases the risk of breast cancer in women under 50, with several observational studies reporting reassuring results [81–83]. For instance, in a Danish Cancer Registry study containing over 70,000 women, the risk of breast cancer was not increased in HRT-treated women aged 40–49 years despite significant increases in those receiving HRT beyond 50 years [83]. Given the much-publicised link between postmenopausal HRT and breast cancer, it is essential women of reproductive age are reassured HRT has not been shown to increase their risk of breast cancer. Accordingly, this cohort does not require enhanced breast cancer screening [84]. HRT is contraindicated in young women with a personal history of breast cancer. In these circumstances, women should be offered non-hormonal treatments to manage symptoms in addition to enhanced screening for cardiovascular risk factors and osteoporosis. Gabapentin and venlafaxine are effective at reducing vasomotor symptoms while oxybutynin can improve sweating related to oestrogen deficiency [85–87]. Neuromodulators are a novel class of medications to treat vasomotor symptoms. Fezolinetant, a recently approved selective neurokinin-3 receptor antagonist, has been shown to reduce the frequency of symptoms [88].

### Testosterone replacement and prostate cancer

There is no convincing evidence linking testosterone replacement in hypogonadal men to an increased risk of *de novo* prostate cancer [89–91]. In the UK Androgen Study, 1,400 men on long-term testosterone replacement underwent regular screening for prostate cancer. One case of prostate cancer was detected for every 212 years of testosterone treatment in the study, comparable to the general population [92]. However, testosterone has been shown to stimulate the growth of metastatic prostate cancer [93]. For this reason, testosterone replacement is contraindicated in patients with prostate cancer and in those who have an unevaluated prostatic nodule or elevated PSA (level > 4 ng/mL). Close discussion is required with urology/ oncology to determine the appropriateness of continuing therapy in low-risk prostate cancer patients [22]. Patients over 40 years old should undergo clinical and biochemical screening for prostate cancer before and 3–12 after commencing testosterone replacement, followed by routine age-related screening thereafter [22].

## Mortality

### Mortality in hypopituitarism

In 1990 Rosen and Bengtsson published their landmark paper which first demonstrated increased mortality in patients with hypopituitarism [4]. These findings were later confirmed in a large prospective study by Tomlinson et al. which reported patients with hypopituitarism had a standardised mortality ratio (SMR) of 1.87 compared to the general population [5]. In both studies mortality was increased in women relative to men [4, 5]. This sex disparity in mortality rates has remained a consistent finding throughout the literature ever since (see Table 3). In a recent meta-analysis, mortality was significantly increased in women, whereas it approached that of the general population in men (SMR 2.09 [95% CI, 1.51–2.89] vs. 1.33 [95% CI 0.95–1.86]) [107]. Cardiovascular disease is the leading cause of mortality in patients with hypopituitarism, accounting for 30–51% of deaths [97, 106]. Women, but not men with hypopituitarism continue to experience increased rates of myocardial infarction, stroke and cardiovascular mortality compared to the general population [6, 102, 106]. In contrast, there is no consistent evidence that respiratory or cancer-related mortality is increased in women relative to their male counterparts. Aside from gender, other factors associated with increased mortality include the underlying pituitary disorder (e.g. Cushing’s disease, acromegaly and craniopharyngiomas), anterior hypopituitarism (gonadotropin and ACTH deficiency and their treatment), diabetes insipidus and treatment modality of pituitary disease (transcranial surgery and radiotherapy). The importance of gonadotropin deficiency was highlighted in a multicentre study by O’Reilly et al. that demonstrated mortality was increased 2.56-fold in individuals with gonadotropin deficiency compared to those with an intact gonadal axis [108]. The study also reported a 2.26-fold increase in mortality in those with ACTH deficiency [108]. Although ACTH deficiency itself is associated with increased mortality, higher doses of hydrocortisone replacement are also associated with cardiometabolic risk factors [109] and cardiovascular mortality [110, 111].

**Table 3:** Studies reporting overall and cardiovascular standardised mortality ratios (SMR) by sex

Study	Etiology of hypo-pituitarism	SMR all-cause	SMR all cause men	SMR all cause women	SMR cardio-vascular	SMR cardio-vascular men	SMR cardio-vascular women
Rosen [4] (1990)	Mixed	1.81	1.47	2.28	1.95	1.7	2.7
Bates [94] (1996)	Mixed	1.73 (1.28–2.28)*	1.50 (1.02–2.13)*	2.29 (1.37–3.58)*	1.35 (0.84–2.07)	1.32 (0.74–2.17)	1.46 (0.5–3.2)
Bulow [95] (1997)	Mixed	2.17 (1.88–2.51)*	1.91 (1.59–2.28)*	2.93 (2.28–3.75)*	1.75 (1.40–2.19)*	1.54 (1.16–2.03)*	2.39 (1.60–3.52)*
Bates [96] (1999)	NFPA	1.2 (0.95–1.55)	1.2	1.3	0.7 (0.5–1.1)	0.9 (0.5–1.4)	0.5 (0.2-1.0)
Nilsson [97] (2000)	NFPA	2.0 (1.9–2.2)*	1.88 (1.72–2.05)*	2.28 (2.04–2.54)*	1.56 (1.4–1.73)*	1.44 (1.26–1.65)*	1.79 (1.50–2.13)*
Tomlinson [5] (2001)	Mixed	1.87 (1.62–2.16)*	1.57 (1.19–2.06)*	2.29 (1.75-3.0)*	1.82 (1.30–2.54)*	1.7	2.34
Svensson [98] (2004)	Mixed	3.8 (3.43–4.19)*	3.36 (2.93–3.38)*	4.54 (3.89–5.26)*	1.4 (1.10–1.75)*	1.20 (0.88–1.60)	1.87 (1.27–2.65)*
Lindholm [99] (2006)	NFPA	1.18 (0.87–1.60)	0.83 (0.55–1.26)	1.97 (1.2–3.21)*	NR	NR	NR
Stochholm [100] (2007)	Mixed	NR	1.90 (1.7–2.2)*	3.4 (2.9-4.0)*	1.9 (1.5–2.3)*	NR	NR
Nielsen [101] (2007)	NFPA	1.21 (0.93–1.59)	0.98 (0.70–1.37)	2.11 (1.35–3.31)*	1.07 (0.59–1.84)	1.07 (0.59–1.93)	1.09 (0.27–4.36)
Olsson [102] (2015)	NFPA	1.06 (0.94–1.19)	0.94 (0.81–1.08)	1.38 (1.12–1.66)*	1.21 (1.06–1.38)*	0.98 (0.78–1.12)	1.66 (1.23–2.18)*
Ntali [103] (2016)	NFPA	3.8 (2.0-4.7)*	NR	NR	NR	NR	NR
van Bunderen [104] (2011)	Mixed	1.29 (1.05–1.59)*	1.01 (0.76–1.35)	1.87 (1.38–2.54)*	1.29 (0.88–1.89)	0.75 (0.42–1.36)	2.67 (1.61–4.44)*
Gaillard [105] (2012)	Mixed	1.13 (1.04–1.24)*	0.94 (0.84–1.06)	1.56 (1.36–1.78)*	0.83 (0.63–1.08)	N.R.	N.R.
Burman [106] (2013)	Mixed	1.42 (1.18–1.70)*	1.33 (1.05–1.66)*	1.63 (1.18 − 2.18)*	1.82 (0.91–3.26)	NR	NR

^Results reported as a SMR with 95% confidence intervals. * Indicates statistically significant result. NR− not reported^

### Mortality and HRT in women

**Fig. 3:**
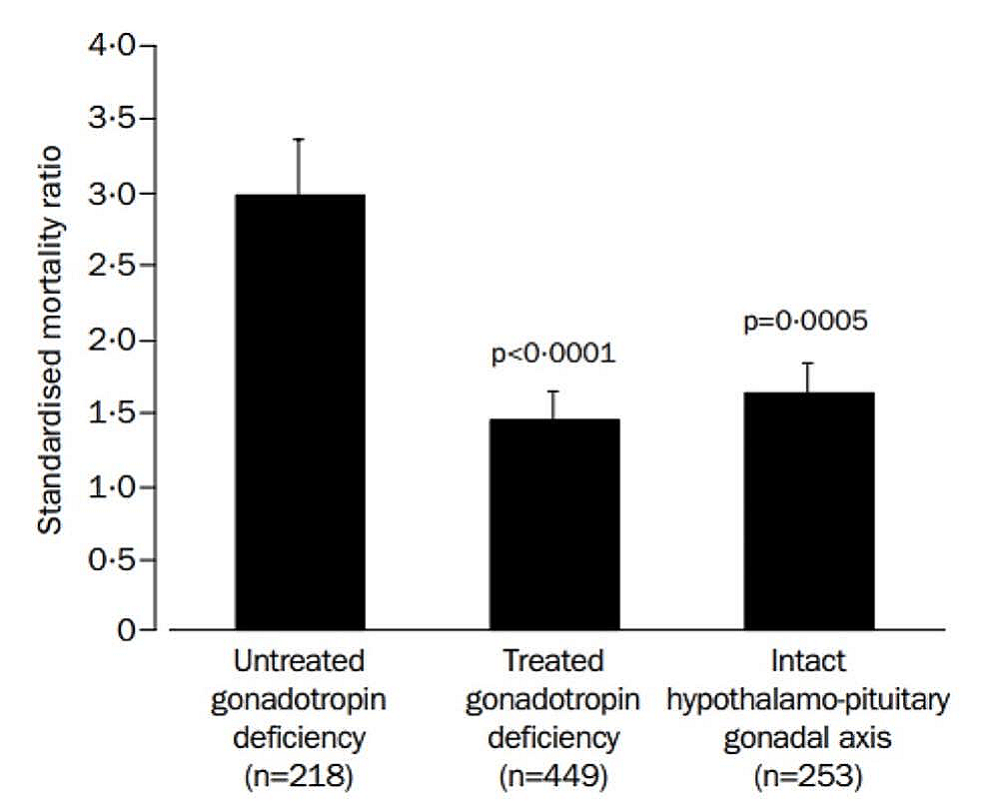
Comparison of SMRs in individuals with untreated gonadotropin deficiency, treated gonadotropin deficiency and an intact gonadal axis. The SMR was significantly higher in those with untreated gonadotropin deficiency compared to those who received sex hormone replacement (SMR 2.97 [99% CI, 2.13–4.13] v’s 1.42 [99% CI, 0.97–2.07], *p* < 0.0001). These results were not analysed by sex. (with permission) [5]

There is limited evidence on the mortality impact of HRT in women with gonadotropin deficiency. Tomlinson et al’s. study reported that gonadotropin deficiency was the only anterior pituitary hormone deficiency to be independently associated with increased mortality [5]. Mortality was significantly higher in patients with untreated gonadotropin deficiency compared to those who received sex hormone replacement (see Fig. 3) [5]. However, the impact of sex hormone replacement on mortality was not analysed by sex. Most other studies are inadequately powered to analyse their results by sex and specific hormone axis. However, a study by Lindholm et al. suggested a mortality benefit from HRT in women with NFPAs. SMRs were higher in those with untreated gonadotropin deficiency compared to women with either treated deficiency or normal gonadotropin function [99]. Given the paucity of data from pituitary cohorts, it is important to examine the impact of HRT in women with POI and early menopause (the latter is defined as the onset of menopause before 45 years), although the limitations in comparison of these patient cohorts need to be recognised. Women with POI and early menopause have an increased risk of coronary heart disease and cardiovascular and overall mortality compared with women who experience menopause after 50 years [112, 113, 114]. Evidence that HRT can reduce these risks is primarily derived from two large cohort studies, the Mayo Clinic Cohort Study of Oophorectomy and Aging and the Nurses’ Health Study [115, 116]. In these studies, women under 50 years who underwent a bilateral oophorectomy but did not subsequently receive HRT had significantly increased all-cause mortality compared to reference populations. In contrast, mortality was not increased in women who received HRT [115, 116]. In the Mayo Clinic study, all-cause mortality was almost 3-fold higher in women who did not receive HRT compared to those who did [116]. In the Nurses’ Health Study, women who did not receive HRT had an 85% increased risk of stroke and 98% increased risk of coronary heart disease compared to HRT-treated women [115]. These findings suggest HRT may play a pivotal role in reducing cardiovascular events and mortality in young women with hypogonadism. Again, the impact of other pituitary hormone deficiencies in this circumstance remains unknown.

### Mortality and testosterone replacement in men

Limited data exists on the impact of testosterone replacement on mortality in men with gonadotropin deficiency. However, the study by O’Reilly et al. observed a trend towards increased mortality in men with NFPAs and untreated gonadotropin deficiency compared to those receiving testosterone replacement (relative risk 1.72 [95% CI 0.71–4.16], *p* = 0.22) [108]. In light of the limited evidence, one can also examine the impact of testosterone replacement in non-pituitary hypogonadal cohorts. Low endogenous testosterone levels in men are consistently associated with increased cardiovascular events and cardiovascular and overall mortality [117–119]. A meta-analysis of 37 observational studies including over 40,000 men reported low endogenous testosterone levels were predictive of cardiovascular and overall mortality with odds ratios of 1.54 [95% CI 1.25–1.89] and 1.26 [95% CI 1.17–1.36], respectively [120]. Despite these findings, the causality of this relationship has not been established. It is important to consider cardiovascular risk factors such as obesity, insulin resistance and diabetes frequently contribute to hypogonadism. Accordingly, testosterone replacement has not been shown to improve mortality in hypogonadal men. A meta-analysis of randomised control trials and observational studies failed to demonstrate that testosterone replacement improved cardiovascular outcomes or overall mortality (hazard ratios 0.87 [95% CI 0.39–1.93] and 0.88 [95% CI 0.55–1.41], respectively) [121].

### Reasons for sex differences in mortality

**Fig. 4:**
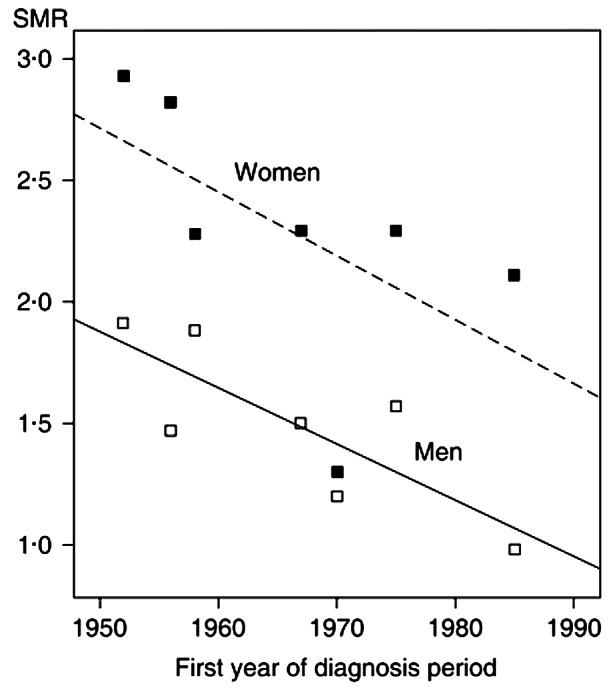
Association between SMR and year of first hypopituitarism diagnosis in individual studies. There was a significant negative correlation between the SMR and year of first diagnosis. When analysed separately by sex, the correlation was significant in men but not women (with permission) [117]

The underlying reasons for the sex disparities in mortality are poorly understood. One possibility is that hypopituitarism simply negates women’s inherent survival advantage over men in the general population. The premature loss of oestrogen’s cardioprotective effect may contribute to the higher prevalence of cardiovascular risk factors, cardiovascular disease and ultimately mortality experienced by women with hypopituitarism. Secondly, inadequate treatment of gonadotropin deficiency in women may contribute towards increased mortality. While adrenal, thyroid and GH deficiencies are replaced at similar rates in both genders, sex hormones are replaced less frequently in women [4, 95, 100]. Estimates suggest almost half of women with gonadotropin deficiency do not receive adequate HRT during their reproductive years [122]. Furthermore, there is evidence that HRT use in women with hypopituitarism has declined in the wake of the WHI [122]. As untreated gonadotropin deficiency (in men and women combined) is an independent risk factor for mortality [5], declining use of HRT may be a contributing factor to increased mortality in women. Thirdly, women and men with ACTH deficiency are typically treated with the same dose of hydrocortisone, meaning women receive higher doses relative to their body surface area [123]. Higher hydrocortisone doses are associated with the development of cardiometabolic risk factors [109]. Therefore, a higher hydrocortisone dose (by body surface area) in women may contribute to the disparities in cardiovascular risk factors and events. Finally, a meta-analysis demonstrated improvements in hypopituitarism-related mortality over time were driven by reduced mortality in men (see Fig. 4) [124]. This suggests women have benefited less from advancements in pituitary care such as pituitary hormone replacement and cardiovascular primary prevention strategies, compared with their male counterparts. It is well-recognised that women in the general population are less likely to receive pharmacological therapy to manage cardiovascular risk factors than men [125, 126]. If this practice extends to women with hypopituitarism it could contribute to adverse cardiovascular events and increased mortality. Clinicians need to be cognisant of the adverse outcomes experienced by women, relative to their male counterparts and the potential biases in the management of their risk factors. Studies are required as a priority to determine if the management of cardiovascular risk factors differs between women and men with hypopituitarism. While the reasons for these disparities remain poorly understood, it is likely that the presence of gonadotropin deficiency, its treatment (or lack thereof) and the management of cardiovascular risks are important contributing factors.

## Conclusion

The risk of morbidity and mortality in patients with hypopituitarism differ markedly by sex. While rates of myocardial infarction, stroke and overall mortality are increased in women with hypopituitarism, they are comparable to that of the general population in men. Despite clear evidence on the beneficial effects and safety of oestrogen replacement in young women with gonadotropin deficiency, many remain undertreated. Barriers to care including concerns about the safety of HRT and clinical inertia around treating cardiovascular risk factors in women must be addressed. Further research is required as a priority to better understand these sex disparities.

## Data Availability

No datasets were generated or analysed during the current study.

## Abbreviations

HPG: Hypothalamic-Pituitary-Gonadal Axis
GnRH: Gonadotropin Releasing Hormone
FSH: Follicle Stimulating Hormone
LH: Luteinising Hormone
NFPA: Non-Functioning Pituitary Adenoma
GH: Growth Hormone
TSH: Thyroid-Stimulating Hormone
ACTH: Adrenocorticotrophic Hormone
CTLA-4: Cytotoxic T-lymphocyte Associated Protein 4
PD-1: Programmed Cell Death protein 1
CHH: Congenital Hypogonadotropic Hypogonadism
HRT: Hormone Replacement Therapy
E2: 17-β Estradiol
EE: Ethinylestradiol
CEE: Combined Equine Oestrogen
OCP: Oral Contractive Pill
HPA: Hypothalamic-Pituitary-Adrenal
CBG: Corticosteroid-Binding Globulin
IGF-1: Insulin-like Growth Factor 1
LDL: Low-density Lipoproteins
TG: Triglycerides
HDL: High-density Lipoproteins
POI: Premature Ovarian Insufficiency
WHI: Women’s Health Initiative
CIMT: Carotid Intima-Media Thickness
BMD: Bone Mineral Density
SMR: Standardised Mortality Ratio
MI: Myocardial Infarction
